# Beyond Tint: Active Ingredient Strategies for Post‐Inflammatory Hyperpigmentation due to Visible Light in Skin of Color

**DOI:** 10.1111/phpp.70112

**Published:** 2026-07-25

**Authors:** Willem Izak Visser, Husna Moola, Susanna Kannenberg, Bianca Tod, Henry W. Lim

**Affiliations:** ^1^ Division of Dermatology, Department of Medicine, Faculty of Medicine and Health Sciences Stellenbosch University Cape Town South Africa; ^2^ Tygerberg Hospital Cape Town South Africa; ^3^ Division of Photobiology and Photomedicine, Department of Dermatology Henry Ford Health Detroit Michigan USA; ^4^ Department of Dermatology College of Human Medicine, Michigan State University East Lansing Michigan USA

**Keywords:** photoprotection, post‐inflammatory hyperpigmentation, skin of color, tinted sunscreen, visible light

## Abstract

**Background:**

Post‐inflammatory hyperpigmentation (PIH) is among the most burdensome sequelae of inflammatory dermatoses in skin of color (SOC), and reduction of visible pigmentation represents the primary clinical outcome of interest. Tinted sunscreens containing iron oxides and/or pigmentary titanium dioxide are currently the best‐evidenced strategy for preventing visible light–driven PIH in SOC. However, cosmetic unacceptability—including white cast, poor shade match, and texture incompatibility with acne‐prone skin—significantly limits real‐world adherence. This concept paper proposes a clinical reframing: when pigment‐based visible light filters are not tolerated, pigmentation reduction may be achievable through proactive topical actives with direct anti‐pigmentation activity.

**Methods:**

This commentary synthesizes the mechanistic and clinical literature on high‐energy visible light (HEVL)–driven pigmentation in SOC, with particular focus on the opsin‐3–MITF–melanogenesis axis and topical actives with demonstrated anti‐pigmentation activity against HEVL‐induced melanogenesis. Evidence is presented to support a proposed clinical reframing and pragmatic algorithm.

**Results:**

HEVL activates opsin‐3 on melanocytes, upregulating MITF and promoting formation of stable, autophagy‐resistant melanosomes—a mechanism distinct from UVA‐driven photooxidation and particularly relevant in darker phototypes. Four prototypical actives with direct clinical or preclinical evidence against HEVL‐induced pigmentation are reviewed.

**Conclusion:**

Pigmentation reduction—rather than the mechanism by which it is achieved—should be recognized as the primary clinical endpoint in photoprotection for PIH in SOC. When pigment‐based visible light filters containing iron oxides and/or pigmentary titanium dioxide are cosmetically unacceptable, a strategy combining broad‐spectrum UVA1 photoprotection with a topical active with anti‐pigmentation activity represents a rational, evidence‐informed alternative that keeps the clinical goal firmly in focus.

When patients with SOC judge therapeutic success, residual pigmentation is often prioritized over inflammatory lesion clearance [1]. Post‐inflammatory hyperpigmentation (PIH) arises as a sequela of diverse inflammatory and injurious stimuli—including acne, eczema, psoriasis, trauma, and cosmetic procedures—and represents one of the most common dermatological complaints in patients with SOC. VL, particularly HEVL, directly exacerbates PIH, making photoprotection a cornerstone of PIH management [2, 3]. In acne‐associated PIH specifically, pigmentation is driven by melanocytic responses to inflammation: arachidonic acid metabolites stimulate melanocyte enlargement, dendricity and tyrosinase activity [4, 5]. *Cutibacterium acnes* may also activate toll‐like receptors on melanocytes and enhance melanogenesis [4]. Most pigment in PIH is epidermal with minimal pigment descent into the dermis, making PIH potentially amenable to topical therapy [5]. Environmental stressors such as sunlight and pollution further amplify melanocyte activity. The South African dermocosmetics consensus statement supports this integrated model of acne suppression, barrier repair and pigment‐directed adjunctive therapy in SOC [6, 7, 8, 9, 10].

Photoprotection is a crucial pillar of proactive hyperpigmentation management and includes photoprotective clothing, wide‐brimmed hats, shade‐seeking behavior, application of the shadow rule and regular sunscreen use [11, 12]. Along with UVB, UVA—particularly long‐wavelength UVA1—and visible light (VL/HEVL) contribute independently to pigmentation [13, 14, 15]. Combined exposure, as occurs under natural daylight conditions, induces a synergistic and more persistent pigmentary response, particularly in SOC [13]. UVA‐induced pigmentation includes immediate and persistent pigment darkening and delayed tanning: early responses are driven predominantly by photooxidation and polymerization of preexisting melanin and melanogenic precursors, whereas delayed tanning reflects more limited de novo melanogenesis [16, 17]. By contrast, high‐energy visible light (HEVL), especially blue light, induces pigmentation through distinct mechanisms. Blue light activates opsin‐3 on melanocytes, enhancing microphthalmia‐associated transcription factor (MITF) activity and promoting the formation of darker, more stable melanosomes that are more resistant to autophagy. This helps explain the intensity and persistence of HEVL‐induced pigmentation, particularly in darker phototypes [13, 18] Conditions such as melasma and PIH are particularly susceptible: clinical studies demonstrate that VL drives relapse and worsening of both conditions, with iron oxide– and pigmentary titanium dioxide–containing tinted sunscreens providing superior pigmentary outcomes specifically through VL attenuation [2, 3]. Oxidation of melanin precursors may play a small role in HEVL‐induced pigmentation [19]. Although both UVA and VL contribute to pigmentation, VL, especially 400–600 nm wavebands, has emerged as especially relevant in SOC, providing the rationale for the present focus on HEVL‐relevant protection [13, 14].

## 1 The Current Standard: Optical Attenuation and Its Adherence Barrier

The most established approach to VL/HEVL‐relevant photoprotection is optical attenuation using pigment‐containing formulations, typically with iron oxides and/or pigmentary titanium dioxide [8, 13]. These systems reduce HEVL transmission and have demonstrated superior pigmentary outcomes compared with non‐tinted organic‐filter sunscreens, aligning with current best practice for pigmentary disorders in SOC [13, 20]. Yet the same mechanism that confers benefit—visible pigment deposition and light scattering—also creates the major barrier to adherence: white or gray cast, poor shade match, transfer and texture dissatisfaction [15]. Micronized or nanoparticulate titanium dioxide improves cosmetic elegance by reducing visible residue, but this also diminishes visible light attenuation, helping explain why elegant untinted inorganic sunscreens do not provide equivalent HEVL protection [8]. Non‐tinted optical filters such as TriAsorB are one effective solution—they extend protection into the visible light spectrum with a peak at 450 nm (HEVL/blue light) [21].

Cosmetic elegance strongly influences sunscreen preference and use [22, 23]. The consequence is a familiar clinical trajectory: improved acne control or partial resolution of the primary dermatosis, persistent dyschromia across a range of inflammatory and pigmentary conditions, patient frustration and erosion of therapeutic confidence.

## 2 A Nonoptical Route to the Same Clinical Endpoint

HEVL is an established driver of pigmentation and has also been implicated in photoaging and photo‐carcinogenesis through oxidative pathways. Non‐filtering photoprotective ingredients, including antioxidants and other photoprotective ingredients (“PINGs”) enhance sunscreen performance across UV‐ and oxidation‐related endpoints [24]. Although antioxidants play a central role in dyschromia care, controlled human studies show inconsistent protection against delayed HEVL‐induced pigmentation. While antioxidant blends may reduce early pigmentation—likely through reduced photooxidation—delayed pigmentation at 7 days still occurs [25]. Topical vitamin E and 7% ascorbic acid failed to prevent HEVL‐induced pigmentation [26, 27]. These findings suggest that antioxidant activity alone is insufficient to interrupt HEVL‐driven melanogenesis, reinforcing the primacy of optical attenuation [25, 26, 27].

PINGs are available in topical and oral formulations. In practice, topical formulations combine multiple active molecules, with efficacy dependent on concentration, stability, and bioavailability. PINGs such as vitamin C, green tea polyphenols, N‐acetylcysteine, and vitamin E play crucial roles in mitigating oxidative stress, inflammation, photoaging, and erythema [24].

The clinical question, therefore, is not whether optical attenuation is superior in principle—it is—but whether a nonoptical strategy can deliver a sufficiently similar pigmentary outcome to justify its use when optical attenuation is not adhered to. Against this background, four topical prototypical molecules with demonstrated activity against HEVL‐induced pigmentation are presented.

1. 2‐Mercaptonicotinoyl glycine (2‐MNG; Melasyl). Among non‐tinted actives, 2‐MNG currently has some of the strongest direct data for HEVL‐induced pigmentation. In a randomized, double‐blind, intraindividual trial, topical 2‐MNG at both 0.5% and 1% significantly reduced HEVL‐induced pigmentation, compared with vehicle [27]. Broader clinical relevance is supported by a 12‐week open‐label regimen study in women with SOC, in which a 2‐MNG‐containing serum plus daily sunscreen improved facial dyschromia [7]. Preliminary comparative data also raise the possibility that a non‐tinted regimen combining strong UVA1 protection with 2‐MNG may, in selected contexts, approximate the pigmentary benefit of tinted pigment‐containing sunscreens [28].
2. Thiamidol. Thiamidol is another promising non‐filtering active. In a randomized intraindividual controlled study, topical 0.2% Thiamidol significantly reduced repeated visible light‐induced pigmentation compared with vehicle [29]. Broader clinical data also support its pigment‐correcting efficacy: in a randomized, double‐blind, vehicle‐controlled trial, 0.2% Thiamidol cream significantly improved facial hyperpigmentation over 12 weeks with good tolerability [30].
3. Fernblock. Botanical extracts such as *Deschampsia antarctica* (EDAFENCE) and *Polypodium leucotomos* (PLE, Fernblock) have shown promise in preclinical models of HEVL injury, including reduction of blue light–induced melanogenesis and modulation of opsin 3‐related pathways [19, 31]. In a randomized controlled study of ten participants exposed to solar light with either an SPF 90 and PLE or SPF 90 alone, a smaller increase in pigmentation was observed in areas with the PLE containing product [32].
4. Niacinamide: In a double‐blind, placebo‐controlled trial, a formulation containing 3% niacinamide showed a trend toward reduced blue light–induced pigmentation compared with placebo, with statistically significant protection against skin reddening [33]. Niacinamide is a commonly added PING to sunscreens [34].

## 3 Similar Clinical Endpoints via Different Mechanisms

The pivotal point is that comparable clinical endpoints may be achievable through different mechanisms. Although visible light attenuation through pigment‐containing formulations or extended spectrum chemical filters is the most direct way to reduce HEVL exposure, the practical goal is reduction of visible pigmentation and prevention of worsening dyschromia—particularly PIH in SOC. If different strategies produce comparable pigmentation outcomes, the approach that is more cosmetically elegant, and therefore more likely to be used consistently, may prove superior in real‐world practice.

This proposition is biologically plausible. If visible light and UVA1 act synergistically to drive pigmentation, then strengthening UVA1 protection while attenuating melanogenesis through a validated biological anti‐pigment active could, in some settings, approximate the pigmentary endpoint achieved by direct HEVL attenuation [13, 24, 27]. When true visible light attenuation is not acceptable or feasible, a regimen combining strong UVA1 protection with targeted pigment suppression may therefore represent a rational alternative route to a similar clinical outcome.

## 4 A Pragmatic Algorithm: “Block what you can; blunt what you can't”

The South African dermocosmetics consensus statement supports a combined approach to PIH in SOC: treat the underlying inflammatory trigger, implement daily broad‐spectrum sunscreen, and incorporate evidence‐based depigmenting adjuncts tailored to tolerance and acne‐prone skin [6]. A visible light‐targeted approach can be viewed in two lanes. The optical lane remains preferred where feasible: broad‐spectrum sunscreen, ideally SPF ≥ 50, together with a tinted iron oxide‐ or pigment‐containing formulation when the patient accepts the shade match, texture and wearability, particularly in pigment‐prone phenotypes and high‐exposure lifestyles [13]. The active ingredient lane serves as an adherence‐promoting alternative: cosmetically elegant sunscreen with robust long‐UVA performance, combined with a HEVL‐induced pigment‐modulating active, selected according to acne activity and barrier sensitivity.

## 5 Limitations

Further research using standardized pigmentation endpoints, commercially available sunscreen formulations (rather than isolated molecules alone), and study populations with active dermatoses such as acne is needed to better define effective, proactive pigmentation care.

## 6 Conclusion

When tinted photoprotection is not cosmetically acceptable, the clinical goal of pigmentation control should not be abandoned—it should be pursued through a different mechanism (see Table 1). Effective VL optical attenuation is not always achievable in routine practice because cosmetic acceptability may limit adherence. In this context, adjunctive pigmentation strategies incorporating agents such as 2‐MNG, Thiamidol, Niacinamide, and Fernblock may represent emerging nonoptical, active ingredient approaches to reducing HEVL‐induced pigmentation. When paired with strong UVA1 protection, these may offer meaningful pigment improvement through a non‐tinted, cosmetically acceptable regimen.

**TABLE 1:** Clinical decision framework for photoprotection and pigmentation management in patients with post‐inflammatory hyperpigmentation (PIH) in skin of color (SOC).

Patient with PIH in SOC requiring photoprotection (acne, eczema, psoriasis, trauma, post a cosmetic procedure)		
Baseline for all patients: 1. Treat the underlying dermatoses 1. Photoprotection package: Sun avoidance, seek shade, wide brimmed hat		
Assess if the patient tolerate a tinted sunscreen containing iron oxide or pigmentary TiO_2_?		
Baseline	**YES**	**NO**
	**Optical Lane**	**Active Ingredient Lane**
	SPF > 50 tinted sunscreen with iron oxide and/or pigmentary TiO_2_ Optimize for shade match and texture for adherence	SPF > 50 sunscreen with robust UVA1 coverage and a topical pigment modulating active Select based on texture and patient preference
Follow up: Assess pigmentary response	If inadequate add adjunctive topical depigmenting agent	
Clinical Endpoint: Reduction of visible pigmentation and prevention of PIH aggravation by primary dermatoses		

Abbreviations: TiO_2_, Titanium dioxide; UVA1, Ultraviolet A1.

## Author Contributions

Willem I. Visser: Conceptualization, writing – original draft, writing – review and editing, and project administration. Husna Moola: Investigation (literature search and data collation), and writing – review and editing. Susanna Kannenberg: Writing – review and editing, and validation. Bianca Tod: Writing – review and editing, and validation. Henry W. Lim: Supervision, writing – review and editing, and validation.

## Funding

This work received no specific grant or financial support from any funding agency in the public, commercial, or not‐for‐profit sectors.

## Disclosure

The authors used ChatGPT (GPT‐5.4, OpenAI) to assist with language editing and outline refinement; the tool was not used to generate scientific content, interpret data, or produce references. All AI‐assisted output was critically reviewed, verified, and edited by the authors, who take full responsibility for the accuracy, integrity, and originality of the manuscript.

## Conflicts of Interest

W.I.V. has previously received honoraria and served on advisory boards for Beiersdorf, NAOS, L'Oréal, Galderma, Genop Healthcare, ISDIN, and Pierre Fabre. H.W.L. has served as an investigator for Incyte, La Roche‐Posay, Pfizer, and PCORI; as a consultant for ISDIN, Beiersdorf, L'Oréal, Eli Lilly, Zerigo Health, Skinosive, Kenvue, Cantabria Labs, NAOS, and Boehringer Ingelheim; and as a speaker at general educational sessions for La Roche‐Posay, Cantabria Labs, NAOS, Pfizer, ISDIN, Clinuvel, and Rxilient. H.M., S.K., and B.T. have no conflicts of interest to declare.

## Data Availability

The data that support the findings of this study are available from the corresponding author upon reasonable request.
